# Reinvestigation of bis­(2,2′-bipyridine)(nitrato-κ^2^ *O*,*O*′)cobalt(III) hydroxide nitrate tetra­hydrate. Corrigendum

**DOI:** 10.1107/S1600536810045472

**Published:** 2010-11-23

**Authors:** A. Wojciechowska, M. Daszkiewicz

**Affiliations:** a Faculty of Chemistry, Wrocław University of Technology, Wybrzeże, Wyspiańskiego 27, 50-370 Wrocław, Poland; b W. Trzebiatowski Institute of Low Temperature and Structure Research, Polish Academy of Sciences, Okólna str. 2, PO Box 1410, 50-950 Wrocław, Poland

## Abstract

The chemical formula in the paper by Wojciechowska & Daszkiewicz [*Acta Cryst.* (2007), E**63**, m2975–m2976] is corrected.

## Abstract 2

Corrigendum to *Acta Cryst.* (2007), E**63**, m2975–m2976.

In the paper by Wojciechowska & Daszkiewicz (2007) ▶, the nitrate ligand should be carbonate and the hydroxide counter-anion should be water, thus making the correct chemical composition [Co(CO_3_)(C_10_H_8_N_2_)_2_](NO_3_)·5H_2_O and the correct chemical name bis­(2,2′-bipyridine)(carbonato-κ^2^ *O*,*O*′)cobalt(III) nitrate penta­hydrate.
